# Relationship between premenstrual syndrome and basic personality traits: a cross-sectional study

**DOI:** 10.1590/1516-3180.2018.0061240418

**Published:** 2018-08-13

**Authors:** Hülya Arslantaş, Filiz Abacigil, Şule Çinakli

**Affiliations:** I PhD. Professor, Department of Psychiatric and Mental Health Nursing, Nursing Faculty, Adnan Menderes Üniversitesi, Aydin, Turkey.; II MD, PhD. Professor, Department of Public Health, Medical Faculty, Adnan Menderes Üniversitesi, Aydin, Turkey.; III MSc. Nurse, Children’s Clinic, Application and Research Hospital, Adnan Menderes Üniversitesi, Aydin, Turkey.

**Keywords:** Premenstrual syndrome, Personality, Adolescent

## Abstract

**BACKGROUND:**

Although many studies have investigated premenstrual syndrome and related factors, there is still only a limited number of studies investigating the relationship between premenstrual syndrome and basic personality traits. This study was conducted to investigate the association between premenstrual syndrome and basic personality traits among university students.

**DESIGN AND SETTING:**

Cross-sectional analytical study conducted in a city in western Turkey.

**METHODS:**

The Premenstrual Syndrome Scale, the Basic Personality Traits Scale and a questionnaire on sociodemographic characteristics developed by the present researchers were applied to 490 female students at the College of Health Sciences of a state university.

**RESULTS:**

Premenstrual syndrome was more common among students living in rural areas (65.1%), students with chronic diseases (74.1%), students who suffered from menstrual cramps (61.1%), students who used cigarettes (72.1%) and students with alcohol intake (65.5%). In the final model of the logistic regression analysis, presence of pain during the menstrual period increased the risk of presence of PMS by a factor of 1.554 (95% confidence interval, CI: 1.033-2.336; P = 0.034) and high scores on the total basic personality traits scale increased it by a factor of 1.016 (95% CI: 1.002-1.030; P = 0.029). The prevalence of premenstrual syndrome was found to be higher among students who were less extrovert (P = 0.007) and less conscientious (P = 0.001); and among students with higher neuroticism (P = 0.000) and negative valance (P = 0.000).

**CONCLUSION:**

This study demonstrates that personality may be associated with premenstrual syndrome.

## INTRODUCTION

The term premenstrual syndrome (PMS) is defined as a set of cognitive and emotional symptoms that start in the luteal phase of the menstrual cycle and swiftly disappear with the start of menstruation. Biological, psychological, physical and social factors have an impact on occurrences of PMS.^1^^,^^2^^,^^3^ Premenstrual symptoms are considered to be affected by increased intensity of menstrual cramps, neurotic personality, increased body mass index, general self-perceived health and cultural differences.^4^^,^^5^

Personality traits play an important role in self-perceived health. Many health problems seen in adolescents have been found to be related to low self-esteem. Adolescents with low self-esteem were found to expect failure, be generally nervous, show less effort towards being successful, ignore important things in life and feel worthless and untalented when they failed.^6^ Personality traits can affect the way in which people perceive daily events. It is known that some stimuli triggered by daily events also trigger PMS in women with sensitive personalities.^7^^,^^8^

However, the literature indicates that the findings from research on the relationship between premenstrual symptoms and some personality traits are complicated.^9^ Gaion and Vieira^10^ demonstrated that individuals with PMS displayed a strong need for performance but a low need for assistance, and also displayed introversion and low desire for change. On the other hand, individuals without PMS showed strong needs for denial and assistance, along with dominance and persistence. Sassoon et al.^11^ found greater percentages of personality disorders (especially obsessive-compulsive personality disorder) among women with severe PMS than among women without PMS. The findings in the literature have indicated that neuroticism and agreeableness were positively related to PMS, while extroversion, conscientiousness, openness to experience and family communication were negatively related to PMS.^12^^,^^13^ In an Iranian study, it was reported that one third of the women who had PMS presented borderline personality traits. Furthermore, the most significant positive correlations were found between PMS and three personality types; neurotic, emotionally stable and introvert.^14^

In the literature, although many studies have investigated PMS frequency, symptoms and related factors,^1^^,^^2^^,^^4^^,^^14^^,^^15^^,^^16^^,^^17^^,^^18^^,^^19^^,^^20^^,^^21^ there is still only a limited number of studies investigating the relationship between PMS and basic personality traits. Personality traits have an important role in coping with health problems. The directions (healthcare applications) needed will affect individuals positively in terms of quality of life. Moreover, no study in Turkey has yet investigated the relationship between PMS and personality traits. Increasing the level of awareness among young women studying nursing will be important, to enable them to provide relevant information to their peers with PMS complaints and provide support through positive coping methods. 

The present study was conducted to investigate and define the factors affecting PMS and the relationship between PMS and basic personality traits, among students at the College of Health Sciences of a state university in Turkey.

## METHODS

### Study design, date, setting and ethical concerns

This cross-sectional study (survey) was conducted during the 2015-2016 spring term in the Nursing, Midwifery, Nutrition and Dietetics Departments of the College of Health Sciences, Aydin, Turkey.

The study was approved by the Ethics Committee of Adnan Menderes University, Aydin (protocol no. 2015/98).

### Participants

Out of the total of 716 female students in the college, 490 students participated: these were students who met the inclusion criteria and who were at school during the study period. No random sampling method was used. Students who did not have any diagnosed psychiatric disorder, declared that they had regular menstrual periods (every 22-35 days), did not have any diagnosed somatic diseases or gynecological or hormonal disorders and were not using any medication or contraceptive pills were included in the study. At the time of enrolling in health-related departments at universities in Turkey, students are required to present a health status document. Therefore, the students’ on-file records were compared with their verbal declarations, and students who were eligible to participate in accordance with the inclusion criteria were checked especially regarding their health status.

### Data collection

The data for this study were collected using a sociodemographic questionnaire and two scales: the Premenstrual Syndrome Scale and the Basic Personality Traits Scale.^22^^,^^23^

The questionnaire consisted of 31 questions that aimed to identify the students’ sociodemographic characteristics and some attributes associated with the menstruation period (age at menarche in years, current menstrual status and menstrual cycle length in days), menstrual problems and management strategies for menstrual problems. The questions were developed based on the literature.^1^^,^^5^^,^^15^^,^^16^

The Premenstrual Syndrome Scale consists of 44 items and has nine sub-dimensions: depressive sensation, anxiety, fatigue, nervousness, depressive thoughts, pain, appetite changes, sleep pattern changes and bloating. The minimum score on the scale is 44 and the highest score is 220. High scores indicate that PMS symptoms are intense. In evaluating the findings from the scale, PMS is considered “present” if the total score exceeds 50% of the maximum score. The Cronbach’s alpha value of the scale has been calculated as 0.75.^22^

The Basic Personality Traits Scale, which was developed by Gençöz and Öncül,^23^ consists of 45 items that are each assessed using a five-point Likert scale. It evaluates six basic personality traits: extroversion, conscientiousness, agreeableness, neuroticism, openness to experience and negative valence. **Extroversion** is assessed using eight adjectives including “timid, withdrawn, shy, talkative, lethargic, enterprising, cold and passive”; **conscientiousness** is assessed using eight adjectives including “self-disciplined, tidy, hard-working, prudent, fussy, determined, irresponsible and lazy”; **agreeableness** is assessed using eight adjectives including “sincere, compassionate, genial, well intentioned, philanthropic, tolerant, sharer and sensitive”; **neuroticism** is assessed using nine adjectives including “nervous, aggressive, angry, temperamental, impatient, capricious, impetuous, touchy and worried”; **openness to experience** is assessed using six adjectives including “self-confident, self-assured, brave, creative, easy going and capable”; and **negative valence** is assessed using six adjectives including “ill-mannered, pretentious, rude, backstabbing, greedy and hidebound”. Higher scores indicate that the person has that personality trait. The Cronbach’s alpha coefficients of the sub-dimensions were as follows: 0.85, 0.74, 0.83, 0.81, 0.65 and 0.69.

### Data analysis

The Statistical Package for the Social Sciences (SPSS) 17.0 for Windows was used for statistical analysis on the data. Descriptive statistics from the study were shown with percentage, mean and standard deviation values. Student’s t test was used for intergroup comparisons of continuous variables and chi-square statistics were used to compare nonparametric categorical variables. Logistical regression analysis was used to determine the possible risk factors that could affect premenstrual syndrome. In this analysis, absence and presence of PMS were taken to be dependent variables, while residence, chronic disease, pain during menstrual period, alcohol consumption, smoking and basic personality traits score were taken to be independent variables. The results from the regression analysis were showed as relative risk (odds ratio, OR) and 95% confidence interval (CI). The backward Wald method was used as the regression model.

## RESULTS

The mean age of the participants was 20.20 ± 1.65 years; 96.6% of the students were single and 67.8% had an income equal to their expenditure. Out of the 490 students included in the study, 26.3% of them lived in rural areas; 48% of their mothers and 33.1% of their fathers were primary school graduates.

53.5% of the students lived in dormitories and 36.7% lived in shared apartments during their education. 11% had chronic diseases other than gynecological problems and 6.3% were continuously using medications. The age at the menarche was found to be 13.2 ± 1.22 years (minimum of 9 years and maximum of 17 years) and the average length of menstruation was 5.4 ± 1.32 days. 71.8% of the students reported that they had menstrual cramps. While 75.5% of the participants viewed menstruation as a natural process of the body, 16.7% of them considered it to be a way for the body to remove dirty blood. 12.7% of the students smoked and 10.8% of them used alcohol. They learned their first information about menstruation from their mothers. The foodstuff that was most consumed among the students before and during PMS was sweets/deserts (79.6%).

The most common symptom among the students that they experienced one week before menstruation was stress-uneasiness, with a rate of 80.6%. The other more common symptoms were breast fullness or swelling, headaches, bloating in the abdomen or “bloating” related to tightness in shoes, clothes or rings or feelings of weight intake, with a rate of 77.3%; and tiredness, lethargy and loss of energy, with a rate of 72.2% (Table 1).

**Table 1:** Symptoms experienced by students one week before menstruation

	n	%
Stress and uneasiness for an unknown reason	395	80.6
Breast fullness or swelling, headache, bloating in the abdomen or “bloating” or feelings of increased weight, experienced with tightness in shoes, clothes or rings	379	77.3
Tiredness, lethargy or loss of energy	354	72.2
Appetite changes manifested through overeating or craving for certain foods	329	67.1
Continuous uneasiness, feelings of anger and increased interpersonal conflicts	309	63.1
Feeling sad, hopeless or worthless	254	51.8
Feeling frustrated or lacking control	220	44.9
Hypersomnia or insomnia	213	43.5
Difficulty in focusing	200	40.8
Frequent desire to cry or bouts of crying	199	40.6
Avoidance of social relations and reduced interest in ordinary events	199	40.6

PMS was seen in 57.1% of the students in this study. No statistically significant relationship was found between occurrences of PMS and income level, parents’ education level, continuous medication use, consumption of coffee, tea and cola drinks, use of oral contraceptives and pain experienced by their mothers or sisters during the premenstrual period. PMS was found to be more common among students who lived in rural areas (χ^2^ = 4.546; P = 0.033); those who had chronic diseases other than gynecological diseases (χ^2^ = 7.104; P = 0.008); those who had menstrual cramps (χ^2^ = 7.909; P = 0.005); those who smoked (χ^2^ = 9.520; P = 0.002); and those who consumed alcohol (χ^2^ = 4.001; P = 0.045) (Table 2).

**Table 2:** Factors affecting occurrences of premenstrual syndrome

Student characteristics	Premenstrual syndrome		No premenstrual syndrome		χ^2^	P
	n	%	n	%		
Residence						
City	196	54.3	165	45.7	4.546	0.033*
Rural area	84	65.1	45	34.9		
Family’s income level						
Income is less than or equal to expenditure	252	58.3	180	41.7	2.112	0.146
Income is more than expenditure	28	48.3	30	51.7		
Mother’s education level						
Elementary school or no education	153	54.8	126	45.2	1.405	0.236
Higher than elementary school	127	60.2	84	39.8		
Father’s education level						
Elementary school or no education	99	55.3	80	44.7	0.388	0.533
Higher than elementary school	181	58.2	130	41.8		
Chronic disease						
No	240	55.0	196	45.0	7.104	0.008**
Yes	40	74.1	14	25.9		
Continuous medication use						
No	257	56.0	201	44.0	5.212	0.074
Yes	23	74.2	8	25.8		
Pain during menstrual period						
No	65	47.1	73	52.9	7.909	0.005**
Yes	215	61.1	137	38.9		
Frequency of drinking coffee						
1-2 cups a week	150	57.0	113	43.0	0.003	0.958
3 or more cups a day	130	57.3	97	42.7		
Frequency of drinking tea						
1-2 glasses a week	39	49.4	40	50.6	2.325	0.127
3 or more glasses a day	241	58.6	170	41.4		
Consumption frequency for cola drinks						
1-2 cans a week	157	56.7	120	43.3	0.056	0.813
3 or more cans a day	123	57.7	90	42.3		
Smoking						
Nonsmoker	218	54	186	46	9.520	0.002**
Smoker	62	72.1	24	27.9		
Alcohol consumption						
No	208	54.7	172	45.3	4.001	0.045*
Yes	72	65.5	38	34.5		
Oral contraceptive use						
No	12	41.4	198	43.0	.027	0.868
Yes	17	58.6	263	57.0		
Premenstrual pain experienced by mother or sister						
No	100	52.1	92	47.9	3.300	0.069
Yes	180	60.4	118	39.6		

*P < 0.05; **P < 0.01.

The prevalence of PMS was found to be higher among the students whose characteristics of extroversion and conscientiousness were of lesser degree (t = 2.701; P = 0.007; t = 3.208; P = 0.001 respectively), and whose neuroticism and negative valence were higher (t = -8.488; P = 0.000; t = -3.607; P = 0.000 respectively). Among the students’ basic personality traits, no relationship was found between agreeableness and openness to experience in terms of the prevalence of PMS (Table 3).

**Table 3:** Relationship between premenstrual syndrome and sub-dimensions of the Basic Personality Traits Scale

	Premenstrual syndrome		No premenstrual syndrome		t	P
	Mean	SD	Mean	SD		
Extroversion	27.35	6.41	28.86	5.78	2.701	0.007*
Conscientiousness	28.67	5.54	30.25	5.20	3.208	0.001*
Agreeableness	33.53	4.25	33.46	4.77	-0.199	0.843
Neuroticism	27.24	0.50	22.40	5.88	-8.488	< 0.001**
Openness to experience	21.26	0.11	21.16	4.09	-0.260	0.795
Negative valence	10.13	3.49	9.04	0.10	-3.607	< 0.001**

SD = Standard deviation. *P < 0.01; **P < 0.001.

In the final model of the logistic regression analysis, it was found that presence of pain during the menstrual period and the basic personality traits score both affected the presence of PMS. Pain during the menstrual period increased the risk of presence of PMS by a factor of 1.554 (95% CI: 1.033-2.336; P *=* 0.034) and high scores on the total basic personality traits scale increased the risk of presence of PMS by a factor of 1.016 (95% CI: 1.002-1.030; P = 0.029).

The most common methods that students used to cope with menstrual cramps included the following: analgesics (36.7%), keeping the abdomen warm (35.5%) and drinking herbal teas (27.3%). These were followed by taking a hot shower (19.6%), lack of continuity at school (16.3%), using intravenous analgesics (10.6%), exercise (9.2%) and going to the emergency department (6.9%).

## DISCUSSION

The findings from this study showed that PMS was seen in 57.1% of the students and that PMS was more prevalent among students who were less extrovert and less conscientious and who had higher neuroticism and negative valance. In this study, the most common complaints that the students had during the premenstrual period were stress, uneasiness, “bloating” or feelings of gaining weight and fatigue or loss of energy. Like in our study, similar complaints such as bloating, fatigue, pain, etc.^1^^,^^24^ have been reported in many other studies. Since these complaints affect students’ social lives, family relations and success at school negatively, it is important to increase awareness of these issues among adolescents and inform them about methods for coping with these complaints.

PMS was more common among the students living in rural areas, students with chronic diseases, patients who suffered from menstrual cramps and students who used cigarettes and alcohol. Pınar and Öncel^25^ and Kebabcılar et al.^26^ reported that smoking was more prevalent among women with PMS in the 15-49 age groups. Other studies conducted in other countries have also reported that smoking and alcohol consumption increase PMS complaints.^27^^,^^28^ Therefore, it is important for future healthcare providers to inform women about the relationship between PMS and smoking/alcohol consumption. Students can be informed about the importance of reducing/quitting smoking and alcohol consumption, increasing their quality of life and exercising to improve health. They can thus become informed about other healthy lifestyles for themselves and for those for whom they provide guidance.

This was the first study to use the Basic Personality Traits Scale in relation to PMS. The findings from studies that have evaluated premenstrual dysphoria in terms of personality traits have indicated the presence of neuroticism. In our study, in terms of personality, the prevalence of PMS was found to be higher among the students who were less extrovert and less conscientious; and among those with higher neuroticism and negative valence. Sassoon et al.^11^ reported that the prevalence of personality disorders among women who had PMS was 27% and, additionally, they found that women with PMS were odd-eccentric, excitable-moody and had anxiety and fears. The most common personality disorder in the PMS group was reported to be obsessive-compulsive personality disorder (18%). Berlin et al.^29^ found that women with PMS showed high levels of histrionic, obsessive-compulsive, self-defeating and dependent personality traits during the follicular and late luteal phases and had higher scores for schizoid, schizotypal, borderline, narcissistic and passive-aggressive personality disorder traits. Similarly, Firoozjaei et al.^14^ reported that the level of borderline traits among women with PMS was 29.9%. They found a positive relationship between neurotic personality, neuroticism, emotional consistency and introversion. Additionally, they claimed that neuroticism accounted for 15% of the severity of PMS.

Some issues relating to women’s health (like PMS) can be perceived differently in different cultures, and this may result in different approaches towards these issues. Especially in developing countries, PMS is not considered to be a health problem and, therefore, awareness about the issue may be insufficient. For this reason, it is important to conduct similar studies in diverse cultures. Moreover, personality is a concept that can be improved through factors such as better education and a better environment, etc. The following support measures can be suggested: participation in social groups for students who are introverts or have low levels of anger control and socialization; improvement of personality structure through using education; organizing of educational programs relating to coping with stress; and directing students to exercise programs and sportive activities.

Our study presents some limitations. It was conducted in only one university and only in its health science departments. This group of subjects can be considered to be a group with a high level of awareness of problem identification and coping methods (or how to seek coping methods). From this perspective, it is possible to suggest that the level of awareness of this problem among adolescents and within society in general may be lower. Therefore, PMS complaints in the general population may be ignored more often. In addition, being unable to reach absent students on the days on which the study was conducted may have had an impact on the study results.

Furthermore, PMS and personality were not evaluated through clinical examinations. Instead, they were evaluated using a scale. The fact that the inclusion criterion of having no psychiatric diagnoses was based on the participants’ self-reports and on information in their medical records was another limitation. Because of stigma and other issues, many people might simply not report psychiatric diagnoses, especially if such conditions are under control and/or not severe; also, some psychiatric diagnoses like depression may be poorly noticed by the patients themselves and depression and psychiatric conditions may be underdiagnosed by physicians. Therefore, we cannot definitively rule out the possibility of psychiatric diagnoses among the participants in this study.

## CONCLUSION

In this study, one out of every two students had PMS, and personality traits had an impact on occurrences of PMS. The basic approach towards health problems is based on selection of patient-specific treatments. Therefore, suitable treatment options based on basic personality traits need to be considered.
